# Sexueller Missbrauch Pflegebedürftiger: Ergebnisse einer bundesweiten Querschnittsstudie bei Hausärzten/-ärztinnen zu Verantwortung und subjektiven Sicherheit im Verdachtsfall

**DOI:** 10.1007/s00391-021-01841-7

**Published:** 2021-01-26

**Authors:** Fabian Moser, Leonhard H. Schütz, Christian Teubner, Nils Lahmann, Adelheid Kuhlmey, Ralf Suhr

**Affiliations:** 1 Stiftung Zentrum für Qualität in der Pflege, Reinhardtstr. 45, 10117 Berlin, Deutschland; 2 Institut für Medizinische Soziologie und Rehabilitationswissenschaft, Charité Universitätsmedizin Berlin, Berlin, Deutschland; 3 Klinik für Geriatrie und Altersmedizin, Charité Universitätsmedizin Berlin, Reinickendorfer Straße 61, 13347 Berlin, Deutschland

**Keywords:** Sexuelle Gewalt, Pflegebedürftigkeit, Hausarzt, Verantwortung, Missbrauch, Sexual violence, Need of care, Family physician, Responsibility, Abuse

## Abstract

**Hintergrund:**

Gewalt gegen pflegebedürftige ältere Menschen ist häufig. Hausärzte/-ärztinnen können bei der Prävention von Gewalt eine Rolle spielen. Insbesondere sexualisierte Gewalt ist stark tabuisiert und wenig untersucht.

**Ziel der Arbeit:**

Ziel dieser Arbeit ist es, die Einstellung von Hausärzten/-ärztinnen zu ihrer Verantwortung bei sexuellem Missbrauch pflegebedürftiger Patienten/Patientinnen zu untersuchen. Zugleich sollen die subjektive Sicherheit hinsichtlich des Vorgehens bei einem Missbrauchsverdacht sowie die Fortbildungsinteressen von Hausärzten/-ärztinnen zum Thema erhoben werden.

**Material und Methoden:**

In einer Querschnittsstudie wurden 1700 Hausärzte/-ärztinnen in Deutschland zwischen September und November 2016 schriftlich befragt. Fragebogen von 302 Ärzten/Ärztinnen konnten ausgewertet werden.

**Ergebnisse:**

Die Unsicherheit hinsichtlich des weiteren Vorgehens bei Verdacht auf sexuellen Missbrauch von pflegebedürftigen Patienten/Patientinnen ist groß. Nahezu alle Befragten sehen es als Teil der ärztlichen Verantwortung, bei sexuellem Missbrauch pflegebedürftiger Patienten/Patientinnen zu intervenieren. Hauptsächliches Fortbildungsinteresse besteht zur Differenzialdiagnose des sexuellen Missbrauchs sowie zum richtigen Vorgehen im Verdachtsfall.

**Schlussfolgerung:**

Fortbildungen, insbesondere zu den Anzeichen sexueller Gewalt gegen Pflegebedürftige, können einen Beitrag leisten, die Handlungssicherheit von Hausärzten/-ärztinnen zu stärken und ihre Bereitschaft zur Prävention zu erhöhen.

## Abstract (en)

**Background:**

Violence against people in need of care is a challenge for long-term care situations. Sexual violence in particular, is subject to strong taboos and has so far been little researched. Family physicians can play a role in preventing violence against people in need of care.

**Objective:**

The objective of this study was to examine family physicians’ attitudes to their responsibilities in cases of sexual abuse of patients in need of care. At the same time, we also examined subjective confidence in relation to the procedure in cases of suspected abuse and family physicians’ interests in further training on this topic.

**Material and methods:**

A cross-sectional study addressed to 1700 family physicians in Germany with a written survey between September and November 2016. Questionnaires from 302 physicians could be evaluated for the study.

**Findings:**

Almost all respondents saw it as part of their responsibility as doctors to intervene in cases of sexual abuse of patients in need of care. There is great uncertainty about how to proceed in cases of suspected sexual abuse of patients in need of care. Respondents’ main interest in further training related to the differential diagnosis of sexual abuse and the correct procedure in cases where abuse is suspected.

**Conclusion:**

Further training provision, particularly on the signs of sexual abuse of people in need of care, could contribute to increasing family physicians’ confidence to act.

## Hintergrund

Die Weltgesundheitsorganisation (WHO) definiert Gewalt gegen ältere Menschen als „eine einzelne oder wiederholte Handlung oder das Unterlassen einer angemessenen Handlung in einer Vertrauensbeziehung, wodurch einer älteren Person Schaden oder Leid zugefügt wird“ ([31], Übersetzung durch die Verfasser). Üblicherweise werden körperliche Gewalt, psychische Misshandlung, Vernachlässigung, finanzielle Ausbeutung und sexueller Missbrauch als Formen von Gewalt unterschieden [19, 25, 32].

Gewalt im Alter ist stark schambehaftet und tabuisiert. Zudem sind potenziell Betroffene oft nur begrenzt erreichbar und auskunftsfähig, z. B. aufgrund kognitiver Einschränkungen [10]. Zugleich kann auch sozial erwünschtes Antwortverhalten zur Unterschätzung des tatsächlichen Auftretens führen [30]. Es liegen daher aktuell nur wenige valide Prävalenzschätzungen vor. Diese unterstreichen jedoch, dass Gewalt gegen ältere Menschen weltweit ein bedeutsames Gesundheitsproblem darstellt [31, 32]. Die Häufigkeit des Auftretens von Gewalt zeigt kulturelle Unterschiede; auch die bevölkerungsbezogenen Prävalenzen der einzelnen Gewaltformen unterscheiden sich deutlich [31]. Psychische Misshandlung ist mit 11,6 % die häufigste Gewaltform. Finanzielle Ausbeutung stellt mit 6,8 % die zweithäufigste Form dar, gefolgt von Vernachlässigung (4,2 %) und körperlicher Gewalt (2,6 %). Sexueller Missbrauch älterer Menschen ist mit 0,9 % seltener.

Die Folgen von Gewalt gegen ältere Menschen sind vielfältig und für Betroffene sowie das Gesundheitssystem bedeutsam. So steigt bei Menschen, die Gewalt gegen sich erfahren haben, beispielsweise das Sterberisiko, sie weisen eine höhere Rate an Depressionen auf, und es kommt vermehrt zu Hospitalisierungen [2, 19, 20].

Pflegebedürftigkeit bzw. physische oder kognitive Einschränkungen sind Risikofaktoren für Gewalt im Alter [25]. Repräsentative Daten zur Prävalenz von Gewalt gegen Pflegebedürftige in Deutschland liegen nicht vor. Verschiedene Studien zeigen jedoch, dass Gewalt gegen Pflegebedürftige in der häuslichen, ambulanten und stationären Langzeitpflege in Deutschland als relevantes Problem wahrgenommen wird und häufig auftritt [7]. Viele pflegewissenschaftliche Untersuchungen befassen sich daher auch mit Ursachen, Auslösern und Möglichkeiten zur Prävention von Gewalt im Kontext Pflege [4]. In bestimmten Fällen, wie bei den freiheitsentziehenden Maßnahmen (FEM), führten diese Bestrebungen zu konkreten pflegerischen Leitlinien und Interventionen zur Gewaltprävention. Sexuelle Gewalt gegen Pflegebedürftige – also die erzwungene Teilnahme einer älteren, pflegebedürftigen Person an einer sexuellen Handlung ohne deren Einwilligung [32] – ist bisher kaum untersucht [3].

Im Zusammenhang mit dem Erkennen und der Prävention von Gewalt gegen Pflegebedürftige sprechen die Breite, Frequenz und Qualität des Arzt-Patient-Kontakts auch für eine mögliche Rolle von Hausärzten/-ärztinnen. So geben über drei Viertel der 60- bis 79-jährigen an, innerhalb der letzten 12 Monate mindestens einmal Kontakt zu ihren Hausärzten/-ärztinnen gehabt zu haben [15]. Die Arzt-Patient-Beziehung ist zudem durch ein Vertrauensverhältnis gekennzeichnet, welches es erlaubt, auch sensible und intime Fragen zu besprechen [8]. Das Potenzial von Hausärzten/-ärztinnen beim Erkennen und bei der Prävention von Gewalt gegen ältere Menschen wird hierbei durch die internationale Forschung gestützt [24]. In einer Studie in Irland wurde gezeigt, dass die meisten Fälle von Gewalt gegen Ältere durch Hausärzte/-ärztinnen in der Häuslichkeit aufgedeckt werden [24]. In einer Befragung von Hausärzten/-ärztinnen in Ohio, USA, gab der Großteil der Befragten an, Hausärzte/-ärztinnen seien besser in der Lage, Gewalt zu erkennen, als andere Gesundheitsprofessionen [16].

Das Thema findet anamnestisch in der Hausarztpraxis jedoch keine systematische Berücksichtigung; eine routinemäßige Befragung älterer Patienten/Patientinnen zu erfahrener Gewalt führen nur jeder/jede siebte Arzt/Ärztin durch [5]; über drei Fünftel befragen diese nie oder fast nie dazu [16]. Auch wird nur ein geringer Anteil der Verdachtsfälle gemeldet [5, 24]. Als ein Grund wird angeführt, dass Ärzte/Ärztinnen sich erst vollständig sicher sein wollten, bevor sie einen Verdacht anzeigten [16]. Knappe zeitliche Ressourcen dürften ebenfalls eine Rolle spielen: Rund zwei Drittel (62 %) der deutschen Hausärzte/‑ärztinnen geben an, nicht ausreichend Zeit für die Behandlung ihrer Patienten/Patientinnen zu haben [13]. Zudem schätzen internationale Studien das ärztliche Wissen zu Gewalt gegen Ältere als gering ein, und es wird auf Fortbildungsbedarf hingewiesen [5, 16].

Für Deutschland liegen keine wissenschaftlichen Befunde zum Wissen von Hausärzten/-ärztinnen oder zu ihrer Rolle beim Erkennen und der Prävention von Gewalt gegen Pflegebedürftige vor. Vor allem gibt es – auch international – keine Untersuchung, die explizit die Frage der von Hausärzten/-ärztinnen wahrgenommenen Verantwortung bei sexuellem Missbrauch pflegebedürftiger Patienten/Patientinnen beleuchtet.

Forschungsfragen dieser Arbeit sind:

1. Wo sehen deutsche Hausärzte/-ärztinnen ihren Verantwortungsbereich beim Erkennen und der Vorbeugung von sexuellem Missbrauch Pflegebedürftiger?
2. Wie sicher sind sich Hausärzte/‑ärztinnen im weiteren Vorgehen bei einem vermuteten Fall von sexuellem Missbrauch Pflegebedürftiger?

## Methodik

### Studiendesign und Vorgehen

In einer bundesweiten Querschnittserhebung wurden Hausärzte/-ärztinnen in Deutschland zwischen September und November 2016 schriftlich befragt^1^. Um eine angemessene Präzision der Konfidenzintervalle zu gewährleisten, wurde eine Teilnehmerzahl von mindestens 250 angestrebt. Ausgehend von einer angenommenen Rücklaufquote von 15–20 %, basierend auf deutschen Erhebungen bei einem vergleichbaren Befragtenkollektiv [18, 22], wurden Fragebogen an 1700 Teilnehmer*innen gesendet. Die Auswahl der Ärzte/Ärztinnen erfolgte per Zufallsstichprobe aus einer kommerziellen Datenbank [1], die nach eigenen Angaben die Daten nahezu aller (99 %) niedergelassenen Arztpraxen in Deutschland beinhaltet. Zur Erhöhung des Rücklaufs wurde 4 Wochen nach Erstversand ein Erinnerungsschreiben, inklusive Fragebogen, an diejenigen geschickt, die bisher nicht teilgenommen hatten. Zur Identifikation des Rücklaufs waren die Fragebogen mit einem adressatenspezifischen Code versehen. Die Studie wurde von der Kassenärztlichen Bundesvereinigung durch einen Artikel im Newsletter *PraxisNachrichten* unterstützt, in welchem die besondere Relevanz hervorgehoben wurde. Ein positives Ethikvotum liegt vor (Eth-21/16).

### Variablen

Erfasst wurden Alter, Geschlecht und Berufserfahrung sowie der Anteil pflegebedürftiger Patienten/Patientinnen. Als „pflegebedürftig“ wurden erwachsene Personen definiert, die aufgrund von körperlichen, kognitiven oder psychischen Beeinträchtigungen dauerhaft Hilfe durch andere benötigen. Die Datenerhebung zu Fragen des sexuellen Missbrauchs wurde im Rahmen einer größeren trinationalen Studie parallel in Deutschland, Österreich und der Schweiz mit dem gleichen Fragebogen durchgeführt; daher wurden Verweise auf nationale sozialrechtliche Regelungen vermieden, und ein expliziter Bezug auf die zum Zeitpunkt der Studiendurchführung gültige deutsche Legaldefinition des SGB XI §14 erfolgte nicht.

Daneben wurde erhoben, ob dem/der Hausarzt/-ärztin bei aktuell versorgten pflegebedürftigen Patienten/Patientinnen bekannt sei, dass diese schon einmal Opfer von sexuellem Missbrauch durch pflegende Personen geworden seien.

Sechs Fragen erfassten die Einstellung zur ärztlichen Verantwortung beim Erkennen und Vorgehen gegen sexuellen Missbrauch Pflegebedürftiger. Sexueller Missbrauch wurde hierbei als „sexuelle Handlungen, die an Menschen ohne deren Einverständnis vorgenommen werden“, definiert.

Zur Messung der Sicherheit hinsichtlich des weiteren Vorgehens im Verdachtsfall wurde die Zustimmung zur Aussage gemessen, dass der/die Befragte „unsicher [wäre], wie [er/sie] weiter vorgehen sollte“, wenn er/sie „den Verdacht hätte, dass ein Patient, der pflegebedürftig ist“ Opfer von sexuellem Missbrauch geworden wäre.

Zum Fortbildungsinteresse wurden zwei Fragen gestellt, die jeweils bejaht bzw. verneint werden konnten. Zunächst wurde das allgemeine Interesse der Teilnehmer*innen an Fortbildung zum Thema des sexuellen Missbrauchs Pflegebedürftiger erfragt. Für Interessierte wurde erhoben, zu welchen einzelnen Themen hierbei Fortbildungsbedarf bestünde.

### Bias

Die Items wurden vor der eigentlichen Studie mithilfe eines mehrstufigen Pretests umfangreich geprüft. In einem ersten Schritt wurde der Fragebogen von Gewaltexperten hinsichtlich Verständlichkeit der Formulierungen und inhaltlicher Vollständigkeit geprüft und angepasst, dann erfolgte durch weitere Experten eine Begutachtung der Validität der gewählten Items. In einer zweiten Phase wurde zur Bestimmung der Retest-Reliabilität und zur Messung sozial erwünschten Antwortverhaltens der vorläufige Fragebogen an eine Zufallsstichprobe von Hausärzten/-ärztinnen versandt. Wurde der ausgefüllte Fragebogen zurückgesandt, erhielt der/die Befragte einen gleichen zweiten Fragebogen. Wurde auch dieser ausgefüllt zurückgeschickt, erhielt er/sie einen Gutschein im Wert von €50, und die Retest-Reliabilitäten der Items dieser abhängigen Datensätze wurden berechnet. Die Retest-Reliabilitäten waren hoch bis sehr hoch. Die Korrelationen mit Maßen sozial erwünschten Antwortverhaltens wiederum waren insgesamt gering. Befragte, welche an den Pretests teilgenommen haben, wurden nicht mehr in der Hauptstudie befragt. Die Vorstudien und ihre Ergebnisse werden an anderer Stelle im Detail beschrieben^2^ [26, 27].

### Datenauswertung und statistische Analyse

Um Adressaten/Adressatinnen, die keine Hausärzte/-ärztinnen sind, von den Analysen ausschließen zu können, wurde erfragt, ob der/die Teilnehmer*in tatsächlich hausärztlich tätig sei. Daneben wurden Fragebogen von der weiteren Auswertung ausgeschlossen, wenn weniger als 80 % der Items beantwortet wurden. Vor der Berechnung deskriptiver Statistiken wurde eine Anpassungsgewichtung auf Grundlage der Informationen des Bundesarztregisters für die Arztgruppen „Allgemeinarzt/-ärztin, praktische Ärztinnen und Ärzte“ sowie „hausärztlich tätige Internisten“ [14] nach Geschlecht und Nielsengebiet [12] vorgenommen. Nielsengebiete resultieren aus einer Zusammenfassung einzelner Bundesländer in 7 in ihrem Konsummuster vergleichbare Gebiete und wurden aufgrund der kleinen Stichprobengröße, statt der einzelnen Bundesländer, für die Gewichtung herangezogen.

Zur Auswertung der gewichteten Daten wurden Mittelwerte und 95 %-Clopper-Pearson-Konfidenzintervalle bestimmt. Zur statistischen Auswertung wurde SPSS® genutzt.

## Ergebnisse

### Rücklauf und Soziodemografie

Die Rücklaufquote beträgt 18,1 %: 302 Fragebogen konnten ausgewertet werden; in 4 Fällen wurden von dem/der teilnehmenden Hausarzt/-ärztin weniger als 80 % der Items ausgefüllt, in weiteren 2 Fällen wurde eine Teilnahme verweigert bzw. abgebrochen. Aus dem Gesamtrücklauf (inkl. der Fälle, die nachweislich nicht oder nicht mehr hausärztlich tätig waren) wurde ein Anteil von 89 % unter den Nonrespondern geschätzt, welcher das Teilnahmekriterium erfüllt. Eine weitergehende Nonresponderanalyse wurde nicht durchgeführt. Der erhobene Rohdatensatz weist bereits ungewichtet eine sehr große Übereinstimmung mit der Grundgesamtheit der deutschen hausärztlich tätigen Ärzte/Ärztinnen auf. Es geben 43,1 % der Befragten an weiblichen Geschlechts zu sein, 56,9 % sind Männer. Das Durchschnittsalter beträgt 55,7 Jahre [54,7; 56,6]. Die Studienteilnehmer*innen geben an, im Durchschnitt 18,6 Jahre [17,5; 19,7] als Hausarzt/-ärztin niedergelassen zu sein. Durchschnittlich 10,9 % [9,8; 12,0] der Patienten/Patientinnen der befragten Hausärzte/-ärztinnen sind pflegebedürftig. Acht Hausärzte/-ärztinnen geben an, von sexuellem Missbrauch bei mindestens einem ihrer pflegebedürftigen Patienten/Patientinnen zu wissen.

### Einstellung der Hausärzte/-ärztinnen zur Verantwortung bei sexuellem Missbrauch

**Figure 1:**
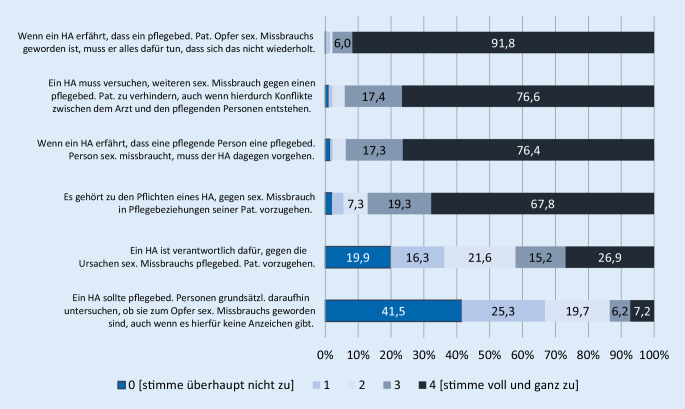

Nahezu alle Befragten (91,8 %) stimmen „voll und ganz zu“, dass Hausärzte/-ärztinnen alles dafür tun müssen, um zu verhindern, dass sich ein sexueller Missbrauch wiederholt (Abb. 1). Dem stimmen noch 76,6 % zu, wenn durch die Intervention Konflikte zwischen Hausärzten/-ärztinnen und den pflegenden Personen hervorgerufen würden. Rund zwei Drittel der Befragten sehen es „voll und ganz“ als hausärztliche Pflicht, gegen sexuellen Missbrauch pflegebedürftiger Patienten vorzugehen; deutlich weniger (26,9 %) sehen sich uneingeschränkt in Verantwortung, gegen dessen Ursachen vorzugehen. Am geringsten fällt die Zustimmung zur grundsätzlichen Untersuchung pflegebedürftiger Patienten/Patientinnen auf sexuellen Missbrauch aus (7,2 %). Es bestehen keine statistisch signifikanten Zusammenhänge zu Strukturvariablen wie Alter, Geschlecht oder Niederlassungsdauer.

### Unsicherheit bezüglich des weiteren Vorgehens

Von den befragten Hausärten/-ärztinnen äußern 26,5 % keine Unsicherheit hinsichtlich des weiteren Vorgehens im Verdachtsfall, weitere 22,7 % äußern geringe Unsicherheit. Die restlichen Befragten stimmen der Aussage in unterschiedlichem Maße zu, bei Verdacht auf sexuellen Missbrauch unsicher bezüglich des weiteren Vorgehens zu sein. Hier zeigt sich ein statistisch signifikanter, jedoch sehr schwach positiver Zusammenhang zwischen der geäußerten subjektiven Sicherheit und dem Alter (r = 0,21, *p* < 0,01), der Niederlassungsdauer (r = 0,19, *p* < 0,01) und dem Geschlecht (r = 0,11, *p* < 0,05) der Befragten.

### Interesse an Fortbildungen zum Thema sexueller Missbrauch

**Figure 2:**
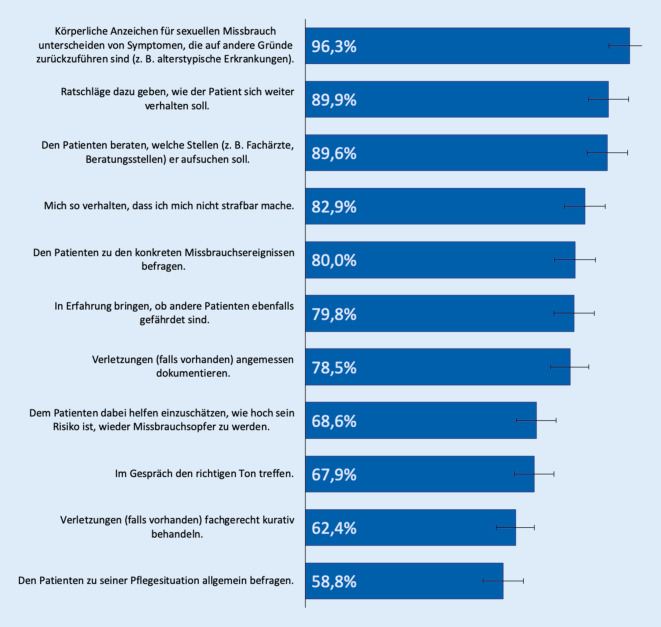

Knapp die Hälfte der Studienteilnehmer*innen (48,2 %) gibt ein Interesse an Fortbildungen zum Thema „sexueller Missbrauch Pflegebedürftiger“ an. Diese Gruppe wurde genauer dazu befragt, welche Fortbildungsinhalte sie hierbei präferiert (Abb. 2).

Fast alle Fortbildungsinteressierten (96,3 % [91,6; 98,7]; *n* = 139) geben an, sich zum Erkennen von Symptomen des sexuellen Missbrauchs weiterbilden zu wollen. Neun von 10 Befragten (89,9 % [83,6; 94,5]; *n* = 134) zeigen Interesse an Fortbildungen zu Ratschlägen, wie der Patient/die Patientin sich weiter verhalten soll bzw. zur Beratung, welche Stellen er/sie aufsuchen soll (89,6 % [83,2; 94,2], *n* = 137). Geringer ausgeprägt ist das Interesse an Fortbildungen zur allgemeinen Befragung von Patienten/Patientinnen zu deren Pflegesituation (58,8 % [50,0; 67,1], *n* = 135).

## Diskussion

In vorliegender Arbeit wurde erhoben, in welchem Maß Hausärzte/-ärztinnen es als ihre Verantwortung ansehen, gegen sexuellen Missbrauch pflegebedürftiger Patienten/Patientinnen vorzugehen. Zugleich wurden die subjektive Sicherheit zum Vorgehen im Verdachtsfall sowie das Weiterbildungsinteresse untersucht.

Nahezu alle Befragten sehen es als Teil der ärztlichen Verantwortung, bei Bekanntwerden des sexuellen Missbrauchs zu intervenieren – selbst auf die Gefahr hin, dass hierdurch Konflikte beispielweise mit den Pflegepersonen entstehen würden. Gegen Ursachen vorzugehen und eine damit stärker präventiv ausgerichtete hausärztliche Verantwortung werden von den in dieser Studie Befragten mehrheitlich verneint.

Sicherlich könnten in dieser Hinsicht auch weitere Gruppen eine wichtige Rolle spielen, wie beispielsweise die Pflegeberatung oder ein Pflegedienst. Durch ihren häufigen regelmäßigen Kontakt zu pflegebedürftigen Personen und dadurch, dass sie in deren körperliche Pflege einbezogen sind, können sehr frühzeitig Anzeichen von Gewalt erkannt werden. Als mögliche Einschränkungen sind hier jedoch zu nennen, dass über zwei Drittel der in der Häuslichkeit lebenden Pflegebedürftigen in Deutschland ausschließlich von pflegenden Angehörigen versorgt werden [28]. Sie haben somit keinen regelmäßigen Kontakt zu professionell Pflegenden. Zugleich kann mitunter von professionell Pflegenden selbst Gewalt ausgeübt werden [4].

Die wenigen vorliegenden internationalen Untersuchungen unterstreichen – allgemein im Kontext der Intervention bei Gewalt gegen Ältere – eine wichtige hausärztliche Rolle [24] und zeigen das hausärztliche Verantwortungsbewusstsein [16] auf. Für eine Verantwortung von Hausärzten/-ärztinnen bei der Intervention gegen sexuellen Missbrauch pflegebedürftiger Patienten/Patientinnen spricht auch die Dichte ihres Patientenkontakts. So sind hausärztlich tätige Allgemeinmediziner*innen die im Erwachsenenalter bei pflegebedürftigen Patienten/Patientinnen am häufigsten aufgesuchten Fachärzte/-ärztinnen [17]. Die meisten Patienten/Patientinnen geben zudem an, ein besonderes Vertrauensverhältnis zu ihren Hausärzten/-ärztinnen zu haben [6], wodurch auch besonders sensible und intime Themen besprochen werden können. Gerade angesichts des starken Tabus des sexuellen Missbrauchs [3] könnte dieses besondere Vertrauensverhältnis in der Hausarzt-Patient-Beziehung die Offenlegung von Missbrauchsereignissen fördern.

Gründe für die mehrheitliche Verneinung einer grundsätzlichen Untersuchung Pflegebedürftiger auf Anzeichen sexuellen Missbrauchs wurden in vorliegender Befragung nicht erfasst. Die Ablehnung könnte in den begrenzten zeitlichen Ressourcen [11, 13] von Hausärzten/-ärztinnen begründet liegen. Hinzu kommt, dass es sich um ein stark tabuisiertes Thema handelt. Auch der Großteil der von Kennedy [16] befragten Hausärzte/-ärztinnen erhebt nie oder fast nie systematisch die von Patienten/Patientinnen erfahrene Gewalt. Weitere Vergleichsstudien zur Einordnung der Ergebnisse liegen aktuell nicht vor.

Die Vorbehalte gegenüber einer Routineuntersuchung könnten darüber hinaus durch Unsicherheit und Wissensdefizite zu den notwendigen weiteren Schritten bedingt sein. Internationale Studien [16, 21] weisen auf Wissenslücken und Fortbildungsbedarf hin. So äußerten beispielsweise in der Studie von McCreadie [23] rund 70 % der befragten Hausärzte/-ärztinnen Fortbildungsbedarf. Für Deutschland gibt es bisher weder Erkenntnisse zum Wissen von Hausärzten/-ärztinnen zu Gewalt gegenüber Pflegebedürftigen im Allgemeinen noch zu Fragen des sexuellen Missbrauchs im Speziellen.

Die Ergebnisse der vorliegenden Untersuchung machen Wissenslücken plausibel. So äußert lediglich ein gutes Viertel der Befragten, hinsichtlich des weiteren Vorgehens bei Verdacht auf sexuellen Missbrauch sicher zu sein. Zugleich gibt knapp die Hälfte der Befragten allgemeines Interesse an Fortbildungen zum Thema an. Von nahezu allen wird Fortbildungsbedarf zu den körperlichen Anzeichen sexuellen Missbrauchs und deren Abgrenzung zu Symptomen anderer Ursache genannt. Zugleich zeigt sich die Unsicherheit auch darin, dass nahezu alle an Fortbildung Interessierten angeben, sich zu Fragen des richtigen Verhaltens bei festgestelltem Missbrauch und zu relevanten Beratungsstellen fortbilden zu wollen.

Tilden et al. [29] konnten zeigen, dass durch Wissensvermittlung und gezieltes Training von Ärzten/Ärztinnen zum Vorgehen bei Gewalt gegen ältere Menschen deren Handlungskompetenz erhöht wurde. In dieser Hinsicht könnten gezielte Fortbildungen einen Beitrag leisten, die bestehende Unsicherheit auch bei deutschen Hausärzten/-ärztinnen zu reduzieren. Vorliegende Arbeit gibt Hinweise auf hausärztlichen Fortbildungsbedarf. Sie hat darin praktischen Nutzen für die Entwicklung von Fortbildungsangeboten, die den inhaltlichen Präferenzen der potenziellen Nutzer*innen entsprechen. Die Entwicklung von Fortbildungsinterventionen und die Prüfung ihrer Wirksamkeit zur Steigerung von Wissen und Handlungskompetenz von Hausärzten/-ärztinnen bedürfen weiterer Forschung.

### Limitationen

Die in der vorliegenden Arbeit erfassten Einstellungen können ein guter Prädiktor für tatsächliches Verhalten sein [9]. Die erhobenen subjektiven Einschätzungen lassen jedoch nur eingeschränkt Aussagen zur tatsächlichen Handlungskompetenz sowie zu vorhandenen objektiven Wissenslücken von Hausärzten/-ärztinnen zu. Trotz umfassender Prüfung und Auswahl der eingesetzten Items [26] könnte sozial erwünschtes Antwortverhalten dazu geführt haben, dass die eigene Sicherheit hinsichtlich des Vorgehens durch die teilnehmenden Ärzte/Ärztinnen tendenziell eher überschätzt wurde. Zudem kann nicht ausgeschlossen werden, dass eher Hausärzte/-ärztinnen mit Interesse am oder Berührung mit dem Thema teilgenommen haben. Hierdurch würden das tatsächliche Wissen von Hausärzten/-ärztinnen in Deutschland und deren Fortbildungsinteresse zum Thema überschätzt. Auch eine fehlende Aktualität der Datensätze der ArztData AG könnte zu einem Selektionsbias geführt haben; dieser erscheint jedoch angesichts des geringen Rücklaufs an unzustellbaren Fragebogen eher unwahrscheinlich. Die hohe Übereinstimmung der Daten der Stichprobe mit der Grundgesamtheit kann als Hinweis auf deren externe Validität gewertet werden, auch wenn abschließend kein absolutes Maß an Repräsentativität genannt werden kann. Aufgrund des Fehlens von Vergleichsdaten aus anderen Studien müssen die Ergebnisse vorliegender Querschnittsstudie durch zukünftige Erhebungen bestätigt und fortgeführt werden.

## Fazit

Die Befragungsergebnisse geben Hinweise auf Unsicherheit und Fortbildungsbedarf von Hausärzten/-ärztinnen zum Thema des sexuellen Missbrauchs Pflegebedürftiger. Aufgrund der besonderen Vertrauensstellung und des häufigen Kontakts zu Pflegebedürftigen stellt sich die hausärztliche Schulung zum Thema als sinnvoll dar. Insbesondere sollten bei Fortbildungsmaßnahmen die Differenzialdiagnose des sexuellen Missbrauchs Pflegebedürftiger sowie das Vorgehen im Verdachtsfall Berücksichtigung finden.

**Fünf Kernaussagen**
1. Hausärzte/-ärztinnen sehen es als ihre Pflicht, bei vermutetem sexuellem Missbrauch eines Pflegebedürftigen zu intervenieren.
2. Eine Routineuntersuchung Pflegebedürftiger auf Anzeichen sexuellen Missbrauchs wird von Hausärzten/‑ärztinnen abgelehnt.
3. Ein Großteil der befragten Hausärzte/-ärztinnen äußert Unsicherheit hinsichtlich des Vorgehens bei Verdacht auf sexuellen Missbrauch Pflegebedürftiger.
4. Die Hälfte der befragten Hausärzte/‑ärztinnen wünscht sich Fortbildungen zum Thema des sexuellen Missbrauchs Pflegebedürftiger.
5. Besonderer Fortbildungsbedarf besteht hinsichtlich der Differenzialdiagnose des sexuellen Missbrauchs und der Abgrenzung von Missbrauchsanzeichen zu alterstypischen Veränderungen.

## Footnotes

1 Schnapp P (2018) Bericht zur ZQP-Studie Gewalt gegen pflegebedürftige Menschen: Prävention durch Hausärztinnen und Hausärzte. Zentrum für Qualität in der Pflege, Berlin, unveröffentlicht

2 Schnapp P (2018) Bericht zur ZQP-Studie Gewalt gegen pflegebedürftige Menschen: Prävention durch Hausärztinnen und Hausärzte. Zentrum für Qualität in der Pflege, Berlin, unveröffentlicht
